# Improved Thermo-Mechanical Properties and Reduced Hydrogen Permeation of Short Side-Chain Perfluorosulfonic Acid Membranes Doped with Ti_3_C_2_T_x_

**DOI:** 10.3390/ma14247875

**Published:** 2021-12-19

**Authors:** Panpan Guan, Jianlong Lei, Yecheng Zou, Yongming Zhang

**Affiliations:** 1 Center of Hydrogen Science, School of Chemistry and Chemical Engineering, Shanghai Jiao Tong University, Shanghai 200240, China; guanpanpan@sjtu.edu.cn (P.G.); ljl2011@126.com (J.L.); 2 Dongyue Future Hydrogen Energy Materials Company, Zibo 256401, China; yechengzou@dongyuechem.com; 3 State Key Laboratory of Fluorinated Functional Membrane Materials, Zibo 256401, China

**Keywords:** short side-chain PFSA, Ti_3_C_2_T_x_ filler, proton conductivity, thermo-mechanical property, hydrogen permeation

## Abstract

Benefiting from its large specific surface with functional -OH/-F groups, Ti_3_C_2_T_x_, a typical two-dimensional (2D) material in the recently developed MXene family, was synthesized and used as a filler to improve the properties of the short side-chain (SSC) perfluorosulfonic acid (PFSA) proton exchange membrane. It is found that the proton conductivity is enhanced by 15% while the hydrogen permeation is reduced by 45% after the addition of 1.5 wt% Ti_3_C_2_T_x_ filler into the SSC PFSA membrane. The improved proton conductivity of the composite membrane could be associated with the improved proton transport environment in the presence of the hydrophilic functional groups (such as -OH) of the Ti_3_C_2_T_x_ filler. The significantly reduced hydrogen permeation could be attributed to the incorporation of the impermeable Ti_3_C_2_T_x_ 2D fillers and the decreased hydrophilic ionic domain spacing examined by the small angle X-ray scattering (SAXS) for the composite membrane. Furthermore, improved thermo-mechanical properties of the SSC/Ti_3_C_2_T_x_ composite membrane were measured by dynamic mechanical analyzer (DMA) and tensile strength testing. The demonstrated higher proton conductivity, lower hydrogen permeation, and improved thermo-mechanical stability indicate that the SSC/Ti_3_C_2_T_x_ composite membranes could be a potential membrane material for PEM fuel cells operating above the water boiling temperature.

## 1. Introduction

Proton exchange membrane (PEM) fuel cells converting the chemical energy of hydrogen to electricity without emission have the potential to replace internal combustion engines for transportation and will play an important role in developing a hydrogen-based energy economy to mitigate the energy dependence on traditional fossil fuels. PEM, which provides the pathways for proton transport and acts as a separation layer for anode fuel (H_2_) and cathode gases (Air/O_2_) is a crucial component determining fuel cell performance [1]. Currently, the most commercially available PEMs are Perfluorosulfonic acid (PFSA)-based materials, with the advantages of their remarkably high proton conductivity under humidified conditions without compromise of mechanical integrity [2], as well as their excellent thermal and chemical stability [3,4]. These exceptional properties of the PFSA membranes stem from their unique phase separation structures comprised of hydrophilic pendant side chains terminated with the solvated sulfonic acid groups and a mechanically stable hydrophobic poly(tetrafluoroethylene) (PTFE) backbone [5,6]. Upon hydration, the dissociated protons can transport through hydrophilic domains of a bicontinuous network of hydrophilic ionic domains and hydrophobic PTFE backbone domains. The early research efforts about PEMs were mostly focused on Nafion membranes with IEC of 0.91–1.00 mmol/g, for which the fuel cell operation temperature was generally limited to 90 °C, restricted by the low glass transition temperature of Nafion (around 110 °C) and to the condition to keep the membrane in hydration for high proton conductivity [7]. To achieve higher proton conductivity and improve thermal stability of the membranes, shorter side-chain PFSAs (compared with the long side-chain in Nafion) have been developed in recent years by Dow, 3M, Asahi Glass, Solvay, Dongyue, etc. The high density of the sulfonic acid groups in the short side-chain (SSC) structure of PFSA membranes enables the superior conductivity, resulting in high fuel cell performance. On the other hand, the shortened side-chains increase the crystallinity and elevate the glass transition temperature, which endow the SSC membranes the potential to be employed in PEM fuel cells operating around 100 °C. Fuel cells operating at a higher temperature can achieve higher power density from the accelerated kinetic reaction rate of the catalyst at elevated temperature and can alleviate the poisoning effects for the catalyst layers. High temperature PEM fuel cells become an emerging technology meeting the requirements in PEM fuel cells for medium-heavy duty transportation applications, however, operation at high temperature brings technical challenges for membrane materials. 

Composite membranes have been demonstrated to be one of the facile and effective approaches to improving the membrane properties for fuel cell applications. Wang et al. reported the composite membranes containing a poly-divinylbenzene (PDVB) in Aquivion (one type of SSC PFSA polymers from Solvay) matrix, with improved mechanical properties and fuel cell power density, as well as the lower hydrogen crossover compared with the recast SSC membrane [8]. Vinothkannan et al. prepared composite membranes with iron oxide (Fe_3_O_4_) nanoparticles anchored over sulfonated graphene oxide (SGO) as a filler in a Nafion matrix, resulting in enhanced proton conductivity, higher water uptake, and the reduced hydrogen permeability due to the interfacial hydrogen bonding between polymer and fillers [9]. Breitwieser et al. also reported on composite membranes with graphene oxide/cerium oxide (GO/CeO_2_) as an interlayer between two layers of Aquivion membrane, for which the MEA showed a low hydrogen crossover current density (1 mA/cm^2^) due to the excellent gas barrier effects of GO sheets [10]. 

MXene, another type of 2D material alongside graphene and its derivatives, has gained wide attention as an additive for composites because of its large electrochemically active surface and excellent mechanical properties. MXenes are obtained by selectively etching A from MAX phase, in which the formula of MAX is M*_n_*_+1_AX*_n_*, where *n* = 1, 2, or 3, “M” is the early transition metal, “A” is mainly a group IIIA or IVA element, and “X” is C and/or N [11]. Ti_3_C_2_T_x_ is one of the typical representatives among the MXene family, and the exfoliated Ti_3_C_2_T_x_ layers are usually terminated with hydroxyl (-OH) and/or fluorine (-F) groups, where T and x represent the terminating groups and their numbers, respectively [12]. There are several reports on the MXenes (Ti_3_C_2_T_x_) composites using different polymers, for example, polyvinyl alcohol (PVA), ultrahigh-molecular polyethylene (UMWPE), epoxy resin, and polypyrrole (PPy) [13,14,15,16]. The specific 2D surface area with the terminated functional groups of -OH or/and -F in MXenes (Ti_3_C_2_T_x_) is also attractive as a promising filler in modifying PEM because it can provide wide and long-range proton transport pathways in membranes compared with other 2D materials [17]. It is reported that incorporating large elastic moduli MXenes (Ti_3_C_2_T_x_) [18] into membranes could reinforce the polymer matrix and improve the thermal stability of composite membranes, as well as the proton conductivity. Fei et al. added Ti_3_C_2_T_x_ into polybenzimidazole (PBI) to prepare a composite membrane for intermediate temperature PEMFC. As a consequence, the composite membrane with 3 wt% Ti_3_C_2_T_x_ (MXene) showed a proton conductivity more than two times higher than that of pristine PBI membrane at the temperature range of 100 °C–170 °C. Both the mechanical properties and thermal stability of PBI/Ti_3_C_2_T_x_ membranes were improved [19]. Zhang et al. prepared the composite PEM by adding the sulfonated Ti_3_C_2_T_x_ as a filler into sulfonated poly (ether ether ketone) (SPEEK) and basic chitosan (CS) matrix to construct proton transporting pathways in membrane for enhancing proton conduction [20]. 

Inspired by the previous enhanced proton conductivity, reduced hydrogen crossover, and higher thermal properties of the MXenes (Ti_3_C_2_T_x_) composite membranes, for the first time in this paper, a different loadings of MXenes (Ti_3_C_2_T_x_) fillers were incorporated into the SSC PFSA polymer to prepare the SSC/Ti_3_C_2_T_x_ composite membranes by the solution casting method. The proton conductivity, water uptake as well as the swelling ratio of the composite membranes were investigated as the function of Ti_3_C_2_T_x_ loading. The effect of Ti_3_C_2_T_x_ on the nanostructure of the composite membranes was examined by SEM and SAXS to explain the hydrogen permeability changing with Ti_3_C_2_T_x_ loading. Finally, the thermo-mechanical properties were measured by DMA and the tensile strength was tested in the temperature range of 80–140 °C to investigate the thermal stability of the SSC/Ti_3_C_2_T_x_ composite membranes for its potential in PEM fuel cell applications operating at above 100 °C.

## 2. Materials and Methods

### 2.1. Materials

The precursor Ti_3_AlC_2_ powder (200 mesh) was purchased from 11 technology Co., Ltd., Jilin, China. The short side-chain (SSC) PFSA polymer sample was supplied by Dongyue Shenzhou New Materials Company Ltd., Zibo, China, without further treatment. The ion exchange capacity (IEC) of the SSC polymer is provided as 1.40 mmol/g (equivalent weight of 714 g/mol) by the supplier. Isopropanol and ethanol were purchased from Sigma-Aldrich Chemical Reagent Co., Ltd., St. Louis, MO, USA and used as received. Hydrochloric acid (HCl) was supplied by Sinopharm Chemical Reagent Co., Ltd., Shanghai, China, and lithium fluoride (LiF) was supplied by Energy Chemical Reagent Co., Ltd., Shanghai, China. Deionized (DI) water (resistance ≥18.2 MΩ·cm) was used throughout the experiments. 

### 2.2. Synthesis of Ti_3_C_2_T_x_ (MXene) Filler from Ti_3_AlC_2_ (MAX)

The Ti_3_C_2_T_x_ was prepared by selectively etching the Al element from Ti_3_AlC_2_ using methods according to previous reports [21,22]. Two synthesis steps were involved: the primary product of Ti_3_C_2_T_x_ and the fewer layer Ti_3_C_2_T_x_.

Primary Ti_3_C_2_T_x_: 

(1) 2.4 g LiF was added into 30 mL 9 M HCl, and stirred at room temperature until the LiF salt was dissolved completely. (2) Gradually 1.5 g Ti_3_AlC_2_ was added to the mixture and stirred for 24 h under 35 °C. (3) The resulting solution was washed by 1 mol/L HCl followed by DI water least three times to remove the residues, and the solid sediment was collected via centrifugation until the pH of the supernatant was close to ~6. (4) We added DI water into the obtained solid sediment from step 3 and treated the solution with ultrasonics in an ice-water bath for 30 min. (5) The suspension of step 4 was centrifuged at 3500 rmp/min for 50 min and the Ti_3_C_2_T_x_ colloid suspension was collected. (6) The primary product (Ti_3_C_2_T_x_) was obtained under cryo-dry for 36 h and was stored at 2–8 °C.

Fewer layer Ti_3_C_2_T_x_: 

(1) The primary Ti_3_C_2_T_x_ was stirred in DI water with the weight ratio of Ti_3_C_2_T_x_:DI water of 1:500 at room temperature for 12 h. (2) The colloidal suspension was ultrasonicated in an ice-water bath for 2–4 h, followed by centrifuging the resultant suspension at 3500 rmp/min for 50 min and collecting the supernatant. (3) The supernatant was filtered using a porous PTFE filter (0.22 μm of pore size) and the solid final product of the fewer layer Ti_3_C_2_T_x_ was dried in an oven at 30 °C for 24 h. 

### 2.3. Fabrication of SSC/Ti_3_C_2_T_x_ Composite Membranes and Pristine SSC Membrane

We dissolved 1.0 g of SSC PFSA polymer in 11.3 g of the isopropanol/water/ethanol (50/45/5 wt%) mixed solvent to form an 8 wt% dispersion. A certain amount of the prepared fewer layer Ti_3_C_2_T_x_ was dispersed into 2.0 g of the same mixed solvent, stirred for 30 min, and ultrasonicated in an ice-water bath for one hour to form the Ti_3_C_2_T_x_ dispersion, which was then added into the SSC dispersion. The obtained mixture was stirred at room temperature for 6 h and sonicated in an ice-water bath for 30 min. The composite membranes were fabricated by casting the dispersions on a glass plate using a doctor blade, dried at 80 °C for 30 min, and annealed at 140 °C for 30 min. After the annealing process, the annealed membranes were quenched to room temperature and peeled off from the glass substrate. The thicknesses of the prepared membranes were in the range of 20 μm to 25 μm measured by a micrometer. The pristine SSC PFSA membrane and the composite membranes are named as SSC and SSC/Ti_3_C_2_T_x_-X where X (X = 0.5, 1.0, 1.5, 2.0, 3.0, 5.0) is the weight percentage of Ti_3_C_2_T_x_ in SSC matrix, respectively.

The pristine SSC membrane was fabricated by the solution casting method as well. SSC PFSA polymer was dissolved into the same mixed solvents as used for composite membrane fabrication (isopropanol/water/ethanol (50/45/5 wt%)) and then casted on a glass plate using a doctor blade. The membrane was dried at 80 °C for 30 min and annealed at 140 °C for 30 min. After the annealing process, the annealed membranes were quenched to room temperature and peeled off from the glass substrate. The thickness of SSC membrane is in the range of 20 μm to 25 μm. 

### 2.4. Characterizations

XRD: X-ray diffraction (XRD) patterns were collected by D8 Advanced X-ray diffractometer (D8 ADVANCE Da Vinci, Bruker Corporation, Billerica, MA, USA) equipped with a Cu Kα radiation. The scanning speed is 5°/min in the 2θ range of 5–70°.

XPS: X-ray photoelectron spectroscopy (XPS, AXIS UltraDLD, Shimadzu Company, Kyoto, Japan) were used to investigate the surface chemical compositions of the fewer layer Ti_3_C_2_T_x_ filler.

SEM: a field-emission scanning electron microscopic (SEM, Nova NanoSEM 450, FEI Company, Hillsboro, OR, USA) was used to observe the morphology of the raw material Ti_3_AlC_2_, the prepared Ti_3_C_2_T_x_ filler and the membranes. The diluted Ti_3_AlC_2_ and Ti_3_C_2_T_x_ suspensions in water were dropped on the silicon wafer to prepare the SEM samples for Ti_3_AlC_2_ and Ti_3_C_2_T_x_. For the cross-section morphology of the composite membranes, samples were immersed into liquid nitrogen for 10 min and then fractured. Distribution of Ti element on the surface of the composite membranes was examined by an energy-dispersive spectrometer (EDS) after SEM observation. 

DMA: thermo-mechanical properties of the SSC and composite membranes were investigated by a dynamic mechanical analyzer (DMA, TAQ800, TA instrument, New Castle, DE, USA). Membrane samples were cut into pieces of 6 mm (width) × 50 mm (length) and tested in a multi-frequency strain mode (tension mode) at a constant frequency of 10 Hz using an initial static force of 0.01 N, a force track of 125%, and an oscillation amplitude of 15 μm. The DMA curves were recorded in the temperature range of 40–200 °C at a heating rate of 10 °C/min.

SAXS: small angle X-ray scattering of the SSC and composite membranes was performed at beamline BL16B1, Shanghai Synchrotron Radiation Facility (SSRF), Shanghai, China. The experiments were carried out at room temperature with X-ray radiation with a wavelength of λ = 1.24 Å (i.e., with energy of 10 keV). The signals were recorded with a Pilatus 2M CCD (172 μm × 172 μm pixel size), and the data were analyzed with the Igor Pro software. 

Mechanical strength: the mechanical properties of the samples were tested using an electromechanical universal testing machine (UTM4102HB, Shenzhen Suns Technology Stock Co., Ltd., Shenzhen, China) equipped with a 100 N load cell. The rectangular samples of 1 cm (width) × 10 cm (length) were stretched at a speed of 20 mm/min. The stretching process was conducted in an oven (WGSY-0200S, Shenzhen Suns Technology Stock Co., Ltd., Shenzhen, China) with controlled temperature. Each sample was kept in the setting temperatures of 80 °C, 100 °C, 120 °C and 140 °C for 20 min before the test. At least five specimens for each membrane sample were tested and the average was calculated for data analysis.

### 2.5. Proton Conductivity

In-plane proton conductivity for the membranes in water was measured using a two-probe conductivity cell connecting on a Zahner electrochemical test system (Zennium 40030, ZAHNER-elektrik GmbH & Co. KG, Kronach, Germany), with the frequency range from 1 to 1 MHz and an oscillating amplitude of 10 mV. The conductivity was calculated from the semi-cycle resistance of Nyquist plot obtained by AC impedance spectroscopy. The membrane samples for proton conductivity at different temperatures were equilibrated for 30 min at the setting temperatures. The calculation of proton conductivity (σ) was according to the following equation:

(1) $$\sigma =\frac{L}{A\times R}$$

where σ is the proton conductivity, mS/cm. *L* is the distance of two electrodes, cm. *R* is the measured ohmic resistance, KΩ, and *A* is the cross-section area of the membrane sample, in cm^2^. 

### 2.6. Water Uptake and Swelling Ratio

Membrane samples for water swelling properties were cut into 2.5 cm × 4.5 cm rectangular pieces. Water uptakes are calculated from the weight of “dry” and “wet” membranes. A “dry” membrane is the dehydrated membrane under vacuum at 90 °C for 12 h. A “wet” membrane is the hydrated membrane which was immersed in DI water for at least 1 hour at the desired temperatures. The “wet” membranes were weighed on a balance within 1 min after the “wet” membranes were taken out from the water and wiped by filter papers to remove excess surface water.

Water uptake and λ (number of water molecules for each SO_3_^−^ group) were calculated by Equations (2) and (3):

(2) $$\mathrm{Water}\;\mathrm{uptake}\;(\%)=\frac{W_{wet}-W_{dry}}{W_{dry}}\times 100\;\%$$

(3) $$\lambda =\frac{n\left(H_{2}O\right)}{n\left(SO_{3}-\right)}=\frac{water\;uptake\;\left(\%\right)\times 10}{18\times IEC}$$

where *W_wet_* and *W_dry_* are the weights of “wet” and “dry” membranes, respectively, in cm; 18 is the molecular weight of water; *IEC* is the ion exchange capacity, in mmol/g.

Swelling ratio (%) was calculated by Equation (4):

(4) $$\mathrm{Swelling}\;\mathrm{ratio}\;\%=\frac{L_{wet}-L_{dry}}{L_{dry}}\times 100\;\%$$

where *L_dry_* and *L_wet_* are the lengths of the “dry” and “wet” membranes, respectively, in cm. 

### 2.7. Hydrogen Permeability

**Scheme 1:**
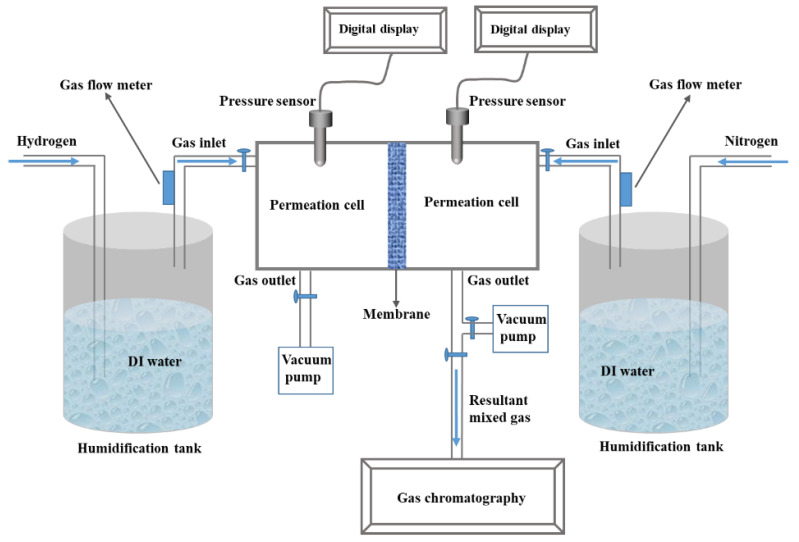
Schematic diagram of hydrogen permeation measurement.

A custom-built apparatus based on the testing method of GB/T 20042.3-2009 standard in China was used to test the hydrogen permeability. The schematic diagram of the apparatus is shown in Scheme 1. The detailed procedures were as follows: firstly, the circular membrane with an effective area of 17.34 cm^2^ was mounted between two permeation cells prior to degassing the cells. Secondly, the permeant gases were introduced to permeation cells with controlled flow rate on both sides (H_2_ and N_2_). The intake pressure of the gases was monitored by the pressure sensor and the gas flow rate was monitored by the flow meters. It was designed to let the gases pass through the DI water tanks at room temperature, for the purposes of roughly controlling the gas flow and humidifying the gases before entering the penetration cells. Finally, the gas sample in the permeation cells of the N_2_ side was collected and analyzed by gas chromatography, after the pressure of the gases on both cells were kept at 101 KPa for 2 hours. The H_2_ content in N_2_ gas sample was calculated based on the calibration curve which was calibrated ahead of time according to the known volume fraction of H_2_ and N_2_ (H_2_:N_2_ = 0:10, 2:8, 3:7, 5:5). At least three specimens of each membrane were tested and the average of the measured hydrogen permeation was reported.

## 3. Results and Discussions

### 3.1. Synthesis of Ti_3_C_2_T_x_ (MXene)

**Figure 1:**
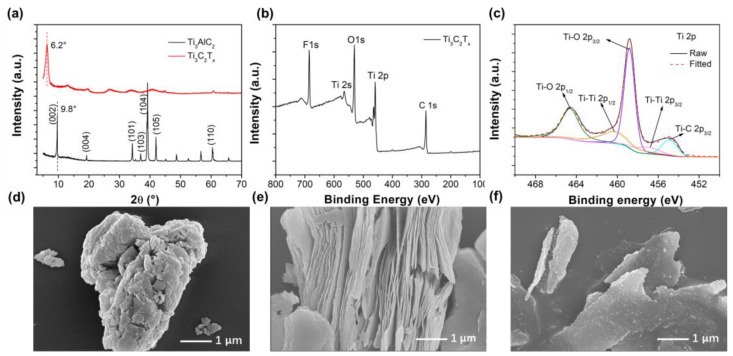
(**a**) X-ray diffraction (XRD) patterns of Ti_3_AlC_2_ and Ti_3_C_2_T_x_; (**b**) X-ray photoelectron spectroscopy (XPS) spectrum and (**c**) deconvoluted high-resolution XPS spectra of Ti 2p for the prepared fewer layer Ti_3_C_2_T_x_. Scanning electron microscope (SEM) images of (**d**) Ti_3_AlC_2_; (**e**) primary Ti_3_C_2_T_x_ and (**f**) fewer layer Ti_3_C_2_T_x_.

The Ti_3_C_2_T_x_ filler was prepared by selectively etching Al layers from Ti_3_AlC_2_ in a mixed solution of LiF and HCl, followed by ultrasonic processing as described in the experimental section. To verify the structure of the prepared Ti_3_C_2_T_x_, XRD patterns of the prepared fewer layer Ti_3_C_2_T_x_ and the raw material Ti_3_AlC_2_ are shown in Figure 1a. The peak (002) shows a downshift of the 2θ in Ti_3_C_2_T_x_ compared to Ti_3_AlC_2_, indicating an increase in the *c*-lattice parameter in Ti_3_C_2_T_x_ [23]. The disappearance of the characteristic peak (104) at 39 °for Ti_3_C_2_T_x_ implies the successful removal of Al layers from Ti_3_AlC_2_ (MAX) phase and the formation of the etched Ti_3_C_2_T_x_ (MXene) [24]. The XPS spectrum of Ti_3_C_2_T_x_ was further collected to verify chemical compositions and oxidization states, shown in Figure 1b, where the signals of O 1s and F 1s suggest the coexistence of Ti–O and Ti–F in Ti_3_C_2_T_x_. High-resolution Ti 2p XPS spectra in Figure 1c disclose the detailed information of the bond stage of Ti. The fitted peaks are related to Ti–O (459.1 eV and 464.6 eV), Ti-C (454.9 eV) and Ti-Ti (461.6 and 457.1 eV) bonds [25,26,27]. The high resolution of O1s from Figure S1a shows the presence of O–H (531.5 eV) and O–Ti (530.0 eV) and the high resolution of F1s from Figure S1b shows the presence of F–Ti (685.8 eV) [27,28]. The results indicate the existence of Ti–O, Ti–OH and Ti–F bonds, suggesting the presence of -O, -OH and -F terminations in Ti_3_C_2_T_x_, for which they are in agreement with previous reports [29]. The morphology features of the Ti_3_AlC_2_ and Ti_3_C_2_T_x_ are examined by SEM and shown in Figure 1d–f. It was found that the raw material Ti_3_AlC_2_ was compact (Figure 1d), while the removal of the Al atom by etching processing resulted a layered “accordion-like” architecture in the exfoliated primary product of Ti_3_C_2_T_x_ (Figure 1e). Figure 1f shows that a fewer-layers structure can be discovered for the final product Ti_3_C_2_T_x_ (MXene), indicating the successful delamination of the Ti_3_C_2_T_x_ fillers after sonication.

### 3.2. Proton Conductivity and Its Activation Energy (E_a_)

**Figure 2:**
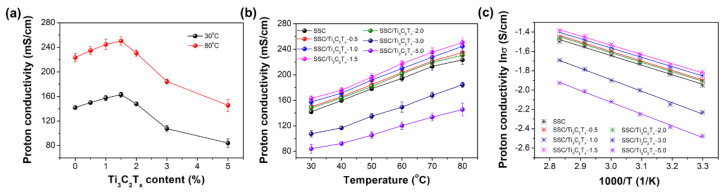
(**a**) Proton conductivity of short side-chain (SSC)/Ti_3_C_2_T_x_ composite membranes in water at 30 °C and 80 °C as a function of Ti_3_C_2_T_x_ content; and (**b**) as a function of temperature; (**c**) Arrhenius plots of proton conductivities.

The proton conductivity of membranes is one of the predominant parameters determining the fuel cell performance. The conductivity of the prepared membranes was measured in water to examine the effects of Ti_3_C_2_T_x_ filler on the proton conductivity. Figure 2a shows the proton conductivity as a function of Ti_3_C_2_T_x_ content at 30 °C and 80 °C in water. The proton conductivity is first increased then decreased as the Ti_3_C_2_T_x_ content increases up to 5%. It reaches the maximum for the SSC/Ti_3_C_2_T_x_-1.5 composite membrane at both 30 °C and 80 °C. The proton conductivity for the SSC/Ti_3_C_2_T_x_-1.5 composite membrane is 163 mS/cm and 251 mS/cm at 30 °C and 80 °C, respectively, which is enhanced by 15% and 12% compared with the values of the pristine SSC membrane (142 mS/cm and 223 mS/cm). The excellent proton conductivity of the SSC PFSA membrane is mainly due to its higher concentration of sulfonic acid groups than that of the long side-chain (LSC) PFSA membranes. For the prepared composite SSC membranes, the hydrophilic functional groups (such as hydroxyl groups) from the addition of Ti_3_C_2_T_x_ could improve the proton transporting environment in membranes and result in a higher proton conductivity. It is reported that these hydrophilic groups can adsorb more water molecules, which solvate the dissociated protons to form ion complexes and further facilitate proton transporting in the vehicle mechanism. On the other hand, the increased water can promote proton hopping in the Grotthuss mechanism [17]. Additionally, the oxygen-containing groups on the Ti_3_C_2_T_x_ surface could facilitate the formation of hydrogen bonding networks for proton transfer pathways [17,19]. However, when the filler content is more than 2.0 wt%, it can be observed that the proton conductivity drops obviously, which could be caused by the aggregation of more Ti_3_C_2_T_x_, as happened in many other composite PEMs [30]. The large amount of the aggregated filler could block the pathways for proton transportation and result in lower proton conductivity for the composite membranes with higher filler contents. It is worth mentioning that those explanations are based on some conclusions from previous reports and not well supported. The mechanisms of the proton transport after incorporation of the Ti_3_C_2_T_x_ filler in the SSC PFSA membrane are not fully understood and need further study.

To investigate the proton transport behaviors of the membranes, the conductivity change as a function of temperature is plotted in Figure 2b. As observed for other PEMs, the proton conductivity of all the prepared membranes in this research is increased when the temperature increases. This is related to the activated water molecules motion as well as the easier motion of polymer chains inside the polymer matrix at the elevated temperature. The Ti_3_C_2_T_x_ filler endows the improved proton conductivity of the SSC PFSA membrane when the Ti_3_C_2_T_x_ content is not more than 2 wt% from 30 °C to 80 °C. The SSC/Ti_3_C_2_T_x_-1.5 composite membrane achieves the highest conductivity. The activation energy (E_a_) for each membrane was calculated using the Arrhenius equation, as reported in references [31,32]:

σ = σ_0_ exp (−E_a_/RT) (5)

σ and σ_0_ are the values of the conductivity and pre-exponential factor, respectively, S/cm, E_a_ is the activation energy required for protons to transport, KJ/mol, R is the gas constant, Jmol^−1^ K^−1^, and T is the absolute temperature, K. 

Figure 2c shows the Arrhenius plots of the conductivity of the membranes. The fitted E_a_ values are 8.23, 8.18, 8.01, 7.95, 8.17, 9.87, and 10.14KJ/mol for the SSC, SSC/Ti_3_C_2_T_x_-0.5, SSC/Ti_3_C_2_T_x_-1.0, SSC/Ti_3_C_2_T_x_-1.5, SSC/Ti_3_C_2_T_x_-2.0, SSC/Ti_3_C_2_T_x_-3.0, and SSC/Ti_3_C_2_T_x_-5.0 membranes, respectively. The activation energy values show the opposite trend as the proton conductivity but with the same turning point at 1.5% in regard to the filler content for the composite membranes. The activation energy decreases with the increase of Ti_3_C_2_T_x_ content (<2.0 wt%) and then starts to increase when the Ti_3_C_2_T_x_ content reaches 2.0 wt%.

It has been well reported [4] that the vehicle mechanism and Grotthuss mechanism are two major proton transport mechanisms for PEMs at a molecular level. In the vehicle mechanism, the protons transport together with water molecules in larger species such as H_5_O_2_^+^ and H_9_O_4_^+^, while in the Grotthuss mechanism, the protons jump from one solvent molecule or functional group to the next by the continuously forming and breaking of hydrogen bonds [32]. In general, activation energy less than 40 KJ/mol is required for proton hopping in the Grotthuss mechanism, while a higher energy (E_a_ > 40 KJ/mol) is required to transport the larger ionic complexes (comparing with protons) in the vehicular mechanism [33,34]. It is assumed that the protons transport preferentially in the Grotthuss mechanism in the investigated membranes of this paper, since the values of the activation energy estimated for the SSC and composite membranes is less than 40 KJ/mol. 

For the composite membranes with less than 2.0 wt% Ti_3_C_2_T_x_ content, the reduced E_a_ values indicate less energy is required for protons to transport through the membrane, because the functional -OH and -F groups from Ti_3_C_2_T_x_ filler enhance the proton transport as discussed in the proton conductivity section above. For the composite membranes with more than 2.0 wt% Ti_3_C_2_T_x_ content, the activation increases as the filler content, indicating more energy is required for proton transport in the membrane.

In summary, the addition of <2.0 wt% Ti_3_C_2_T_x_ in the membrane could improve the proton conductivity of the SSC membrane, while the addition of >2.0 wt% Ti_3_C_2_T_x_ in the membrane has deleterious effects on conductivity; 1.5 wt% of Ti_3_C_2_T_x_ is considered as an optimized content of the SSC/Ti_3_C_2_T_x_ composite membranes for proton conductivity. Considering the much lower conductivity of the SSC/Ti_3_C_2_T_x_-5.0 composite membrane, the other properties of this membrane will not be discussed in the next sections.

### 3.3. Water Uptake and Swelling Ratio

**Figure 3:**
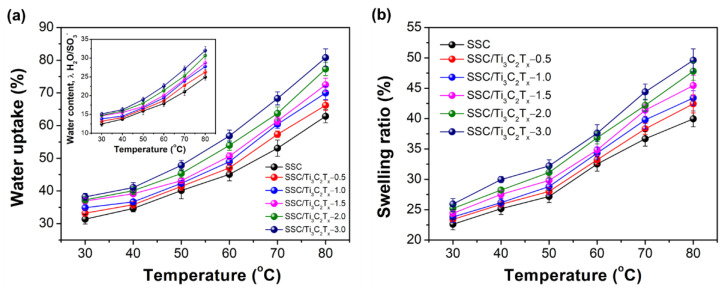
(**a**) Water uptake (the inserted figure is the water contents of membranes, λ) and (**b**) swelling ratio of the membranes in water as a function of temperature.

The water uptakes and swelling ratios in length direction of the fully hydrated membranes in liquid water at different temperatures were measured and are shown in Figure 3a,b. It can be observed that the water uptake increases as the function of temperature and the water uptakes of all the composite membranes are higher than the pristine SSC membrane, increasing gradually as the increase of Ti_3_C_2_T_x_ content. The water uptakes are 31.4% and 36.9% for the SSC membrane and the SSC/Ti_3_C_2_T_x_-1.5 composite membrane at 30 °C, while the values are 62.8% and 72.4% at 80 °C. The higher water uptake for the composite membranes would be mainly attributed to the augmented hydrophilic ionic domains in the membranes from the hydrophilic -OH groups on the surface of the added Ti_3_C_2_T_x_ [17]. Thus, the water uptake increases as the content of Ti_3_C_2_T_x_. The water contents, λ, at different temperatures are shown in the inserted figure of Figure 3a, for which the trend is consistent with that of the water uptakes in Figure 3a. High water uptake can improve the proton conductivity normally, while it also lowers the dimensional stability. Similarly, the swelling ratios of the composite membranes all increase with the temperature and with the filler content. The swelling ratios are 22.5% and 24.4% at 30 °C for the SSC membrane and the SSC/Ti_3_C_2_T_x_-1.5 composite membrane, while they are 39.9% and 45.4% at 80 °C. The higher swelling ratio for the composite membranes is related to the higher water uptake, which is difficult to mitigate for most of the PEMs.

### 3.4. Small Angle X-ray Scattering (SAXS)

The nanostructure of the SSC and SSC/Ti_3_C_2_T_x_-1.5 membranes in dry state was investigated by SAXS to further understand the influence of Ti_3_C_2_T_x_ filler on the nanostructure of the composite membranes. Information related to the organized nanostructure in the membranes can be extracted from the different q-ranges. For PFSA polymers, the peak with q range of 0.02 Å^−1^ < q < 0.1 Å^−1^ for the inter-crystalline spacing of 10–25 nm (based on the equation of d = 2π/q, wherein, d is the domain spacing and q is the scattering vector) represents the spacing between the PTFE crystalline domains of the polymer matrix (so-called matrix knee). Another characteristic peak with the q range of 0.1 Å^−1^ < q < 0.3 Å^−1^ represents the mean correlation distance between the ionic domains (3–6 nm) (so called ionomer peak) [3]. 

**Figure 4:**
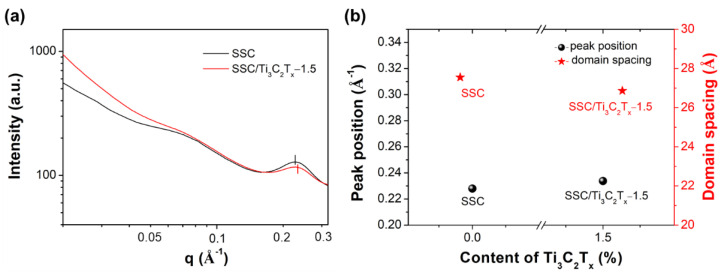
(**a**) 1D small angle X-ray scattering (SAXS) profiles of SSC and SSC/Ti_3_C_2_T_x_-1.5 membranes; (**b**) the Gaussian fitting results of SAXS profiles for ionic phase peaks (the peak between 0.2 to 0.3 Å^−1^) of SSC and SSC/Ti_3_C_2_T_x_-1.5 membranes.

Figure 4a shows the scattered intensity as a function of the scattering wave vector (q). For both membranes, the positions of lower q peaks (~0.07 Å^−1^) which are related to the PTFE crystalline structure [35] almost remain constant, indicating the addition of fillers have less impact on the ordering of the PTFE backbones. These low q peaks are much broad and not as obvious as the ionomer peak, indicating less periodic ordering in the crystalline domains or low crystallinity. However, the position of the higher q peak (ionomer peak) is observed differently in Figure 4a for the SSC/Ti_3_C_2_T_x_-1.5 composite membrane compared with the SSC membrane. It shows the changes for the fitted peak position and domain spacing in Figure 4b. The ionomer peak position shifts to higher q, from 0.228 Å^−1^ for the pristine SSC membrane to 0.233 Å^−1^ for the composite membrane of at 1.5 wt% filler content, while the domain distance decreases from 27.5 Å to 26.9 Å. The reduced domain spacing could be attributed to the hydrophilic functional groups on the surface of Ti_3_C_2_T_x_ which enhance the interfacial interaction between the Ti_3_C_2_T_x_ and the SSC polymer chains. Similar effects have been reported by other groups with the addition of 2D fillers or inorganic particles in PFSA polymers. For example, Li et al. doped different sizes of graphene oxide (GO) sheets to the Nafion matrix and reported that all the composite membranes showed a smaller domain distance compared with the recast Nafion, because of the interfacial oxygen groups interacting with the Nafion chains [36]. 

### 3.5. Scanning Electron Microscopy (SEM) Morphology

**Figure 5:**
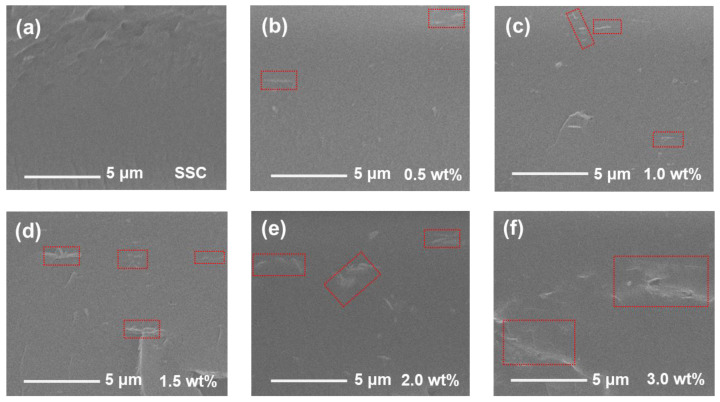
Cross-sectional SEM images of the membranes. (**a**) SSC; (**b**) SSC/Ti_3_C_2_T_x_-0.5; (**c**) SSC/Ti_3_C_2_T_x_-1.0; (**d**) SSC/Ti_3_C_2_T_x_-1.5; (**e**) SSC/Ti_3_C_2_T_x_-2.0; and (**f**) SSC/Ti_3_C_2_T_x_-3.0.

SEM was employed to investigate the surface morphology in the cross-section of the membranes, shown in Figure 5. The areas marked by the red rectangles represent the Ti_3_C_2_T_x_ fillers in the SEM images. The cross-section of the pristine SSC membrane is dense and uniform except for a few stripes caused by fracturing (Figure 5a), and no filler can be observed. There are fewer fillers in the polymer matrix with 0.5 wt% Ti_3_C_2_T_x_ (Figure 4b) compared with other composite membranes with the higher Ti_3_C_2_T_x_ contents. The Ti_3_C_2_T_x_ fillers in the cross-section SEM images gradually increase as the Ti_3_C_2_T_x_ content increases and the size of the Ti_3_C_2_T_x_ fillers is less than 5 μm when the Ti_3_C_2_T_x_ loading is less than 2.0 wt%. It can be observed that when the Ti_3_C_2_T_x_ loading is more than 2.0 wt%, the Ti_3_C_2_T_x_ fillers start to aggregate, shown in Figure 5f of the SSC/Ti_3_C_2_T_x_-3.0 membrane. The aggregation of Ti_3_C_2_T_x_ could result in a reduced active surface area of the hydrophilic functional groups and affect the membrane property. Furthermore, the aggregation would cause structural defects such as cracks and pinholes, which will result in poor membrane durability and could cause higher gas permeability. The aggregation of Ti_3_C_2_T_x_ might be one of the reasons that resulted in much lower proton conductivity for the SSC/Ti_3_C_2_T_x_-3.0 composite membrane (Figure 2a,b) compared with the SSC membrane.

**Figure 6:**
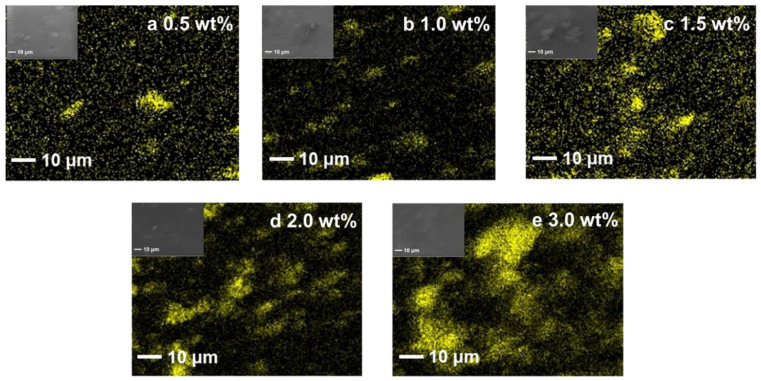
Energy-dispersive spectroscopy (EDS) of Ti element distribution for the SSC/Ti_3_C_2_T_x_ composite membranes.

The distribution of titanium element (Ti) on the surface of the membrane was further examined by an energy-dispersive spectrometer (EDS) to investigate the distribution of the fillers in the membranes, shown in Figure 6. It can be seen that the Ti in the membranes becomes denser when the Ti_3_C_2_T_x_ loading is above 1.5 wt%, indicating the Ti_3_C_2_T_x_ starts aggregating in the composite membranes with Ti_3_C_2_T_x_ contents higher than 1.5 wt%. This phenomenon agrees with the SEM results of the cross-sectional morphology in that the aggregation of Ti_3_C_2_T_x_ fillers occurs when the Ti_3_C_2_T_x_ content is higher than 1.5 wt%. 

### 3.6. Hydrogen Permeation

**Figure 7:**
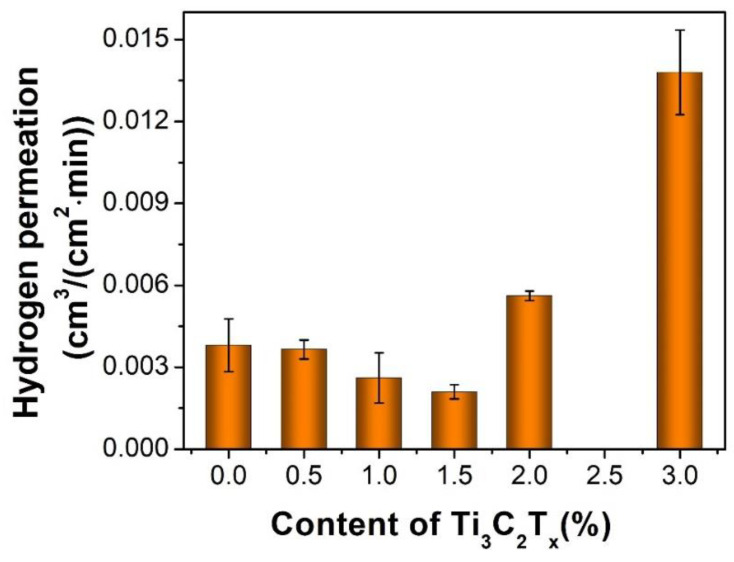
Hydrogen permeation of the membranes with different Ti_3_C_2_T_x_ contents at room temperature.

The smallest size atom of hydrogen in a fuel cell will cross over from the anode to the cathode through the dense polymer membrane. H_2_ permeation is an important parameter for membranes in PEM fuel cells since hydrogen crossover reduces the fuel efficiency and causes safety concerns as well. The H_2_ permeation for the pristine SSC and composite membranes was measured by an apparatus with the diagram shown in Scheme 1. The concentration of H_2_ was measured by gas chromatography. The higher H_2_ permeability indicates the higher H_2_ crossover through the membrane, therefore low H_2_ permeability is expected for membranes for applications of PEM fuel cells. The H_2_ permeation of the composite membrane gradually decreases with the Ti_3_C_2_T_x_ filler content up to 1.5wt%, then increases rapidly with the higher filler content (Figure 7). The minimum H_2_ permeation of ~2.1 × 10^−3^ cm^3^/(cm^2^ min) is observed for the SSC/Ti_3_C_2_T_x_-1.5 composite membrane, which is 45% less than ~3.8 × 10^−3^ cm^3^/(cm^2^ min) for the pristine SSC membrane. The significant reduction of the SSC/Ti_3_C_2_T_x_-1.5 composite membranes in H_2_ permeation could be ascribed to the incorporation of the impermeable Ti_3_C_2_T_x_ filler as the same effects in the GO composite membranes. It is reported that up to two orders of magnitude lower permeation for hydrogen diffusing through the GO filler (another type of 2D materials) compared through the Nafion membrane and that the impermeable GO fillers can reduce the hydrogen crossover for GO composite membranes [10]. Vinothkannan et al. also reported that the prepared Nafion/GO composite membranes showed a reduction of H_2_ permeation compared with the pristine Nafion, due to an increased tortuous path for the diffusion of H_2_ gas molecules after incorporating the GO as a filler [9]. Similar to GO fillers, the Ti_3_C_2_T_x_ fillers could block the hydrogen permeating pathways, resulting in lower hydrogen permeation. In addition, the reduced hydrogen permeation of the SSC/Ti_3_C_2_T_x_-1.5 composite membrane could also be explained by the decreased ionic domain spacing for that membrane examined by SAXS. It is reported that hydrogen cross-over is predominantly through the hydrophilic ion clusters and their interface with PTFE domains for PFSA membranes [3]. A report by Dorenbos et al. indicated that the gas permeation decreased with the domain distance of the hydrophilic domains in a Nafion membrane [37]. In this research, the ionic domain spacing decreased from 27.5 Å^−1^ to 26.9 Å^−1^ for the pristine SSC membrane compared with the SSC/Ti_3_C_2_T_x_-1.5 composite membrane by SAXS results shown in Figure 4. The narrowed spacing among the ionic clusters also increases the tortuosity of hydrogen pathways resulting in lower hydrogen permeation. On the other hand, when the filler content is above 2.0 wt%, it is found that the H_2_ permeation of the composite membranes increased significantly. As suggested by SEM images in Figure 5, the large size aggregation of the fillers is formed for the composite membranes with the filler content over 2 wt%. It is hypothesized that the high H_2_ permeation could be associated to the defects or micro-pores developed by these larger size fillers, through which the H_2_ gas diffuses easily. It is worthwhile mentioning that both H_2_ permeation increasing and proton conductivity dropping occur in filler content over 2 wt% for the composite membranes. The filler content of 1.5 wt% could be the turning point for the property changes of the SSC/Ti_3_C_2_T_x_ composite membranes in this study.

### 3.7. Dynamic Mechanical Analyzer (DMA)

**Figure 8:**
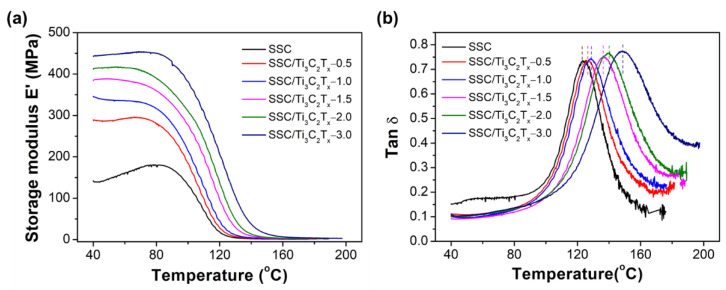
(**a**) Storage modulus and (**b**) tan δ curves of the membranes measured by dynamic mechanical analyzer (DMA).

Thermo-mechanical properties of the pristine SSC and composite membranes were studied by DMA to investigate the filler–polymer interactions on the membrane’s thermal behaviors. As shown in Figure 8a, the value of storage modulus (E′) is enhanced with the fillers content. The storage modulus for the SSC/Ti_3_C_2_T_x_-1.5 composite membrane is ~385 MPa at 40 °C, which is almost 2.7 folds higher than that of the SSC membrane (~140 MPa). Storage modulus reflects one elastic property for polymers. It indicates the interactions between the surfaces of the Ti_3_C_2_T_x_ filler and the SSC polymer matrix. The ratio of storage modulus (E′)/loss modulus (E″, not shown in the figure) represents tan δ, which is another parameter to evaluate the polymer relaxation behavior. The peak temperature in a tan δ curve, represented as the alpha-relaxation temperature (Tα), is attributed to the primary relaxation process that reflects the glass transition temperature (Tg) for most ionic polymers. Tα is believed to be assigned to the beginning of long-range mobility of both polymer backbone and side chains, which is caused by lowering the electrostatic interactions within ionic clusters [38] or caused by the destabilization of an electrostatic network [39]. It can be observed that the Tα of the composite membranes with the Ti_3_C_2_T_x_ filler are higher than that of the pristine SSC membrane, and it shifts to higher temperature as the filler content increases. The Tα of the pristine SSC membrane is 123 °C, while the Tα of the composite membranes increases from 126 to 149 °C with the increase of the filler content from 0.5 wt% to 3.0 wt%. The enhanced storage modulus and Tα are attributed to the hydrogen bonding interactions between the functional -OH/-F groups on the surface of Ti_3_C_2_T_x_ fillers and the SSC polymer chains [17]. The hydrogen bonds formed restrict the reorganization of polymer chains and stiffen the membranes, resulting in the higher Tα for the composite membranes. Furthermore, the higher Tα could also be associated with the reinforcing effect by the high storage modulus nature of Ti_3_C_2_T_x_ filler in the composite membranes. The thermal stability of composite membranes is improved by incorporating Ti_3_C_2_T_x_ filler into the SSC membranes. As a result, the improved thermal stability could enable the use of SSC/Ti_3_C_2_T_x_ composite membranes in PEMFC applications operating at higher than 100 °C conditions.

### 3.8. Mechanical Strength

**Figure 9:**
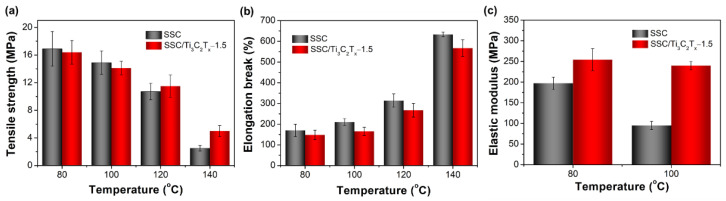
Mechanical properties of SSC and SSC/Ti_3_C_2_T_x_-1.5 composite membranes under different temperatures. (**a**)Tensile strength and (**b**) elongation break of membranes under 80 °C, 100 °C, 120 °C and 140 °C; (**c**) elastic modulus of membranes under 80 °C and 100 °C.

The stress–strain properties of the pristine SSC membrane and the optimized SSC/Ti_3_C_2_T_x_-1.5 composite membrane were tested to further understand the effects of the filler on the mechanical property of the composite membranes. Knowing Tα reflects the glass transition temperature (Tg) of polymers, and the Tα is 123 °C for the SSC membrane and 136 °C for the SSC/Ti_3_C_2_T_x_-1.5 membrane, both membranes were tested for tensile strength under four temperatures: 80 °C, 100 °C, 120 °C, and 140 °C. Three temperatures that are lower than Tg and one temperature that is higher than Tg were selected to investigate the membrane’s tensile strength near the Tg of the polymer membranes. The values of tensile strength, elongation break, and elastic modulus of the membranes obtained by stress-strain curves are shown in Figure 9. It can be seen that the tensile strength reduces while the elongation increases as the temperature increases from 80 to 140 °C for both membranes, which indicate that the polymer matrix become “softer”. This is expected for polymer materials since the higher temperature will provide more energy promoting the motivation of polymer chains in the matrix of the membrane, which further reduces stress and enhances elongation break when stretching the membrane. Besides, the tensile strength shows a dramatic decrease at 140 °C because the temperature is higher than the Tg of both membranes. The tensile strength of SSC/Ti_3_C_2_T_x_-1.5 composite membrane is slightly lower than that of the SSC membrane at 80 °C and 100 °C. However, the tensile strength of the SSC/Ti_3_C_2_T_x_-1.5 composite membrane becomes stronger than that of the SSC membrane at above 120 °C. This is because 120 °C is very close to the glass transition temperature (Tg ≈ Tα = 123 °C) of the SSC membrane, for which the polymer chains become flexible and the SSC membrane is soft at 120 °C. When the testing temperature reaches 140 °C, the improvement in tensile strength for the composite membrane is significant when compared to the values of the SSC/Ti_3_C_2_T_x_-1.5 composite membrane and the SSC membrane at 140 °C. The tensile strength of the SSC/Ti_3_C_2_T_x_-1.5 is 7% higher than that of the SSC membrane at 120 °C (11.5 MPa versus 10.7 MPa), while almost twice as strong at 140 °C (5.0 MPa versus 2.5 MPa). Again, the improved tensile strength of the SSC/Ti_3_C_2_T_x_ composite membrane at 120 °C and 140 °C demonstrates the potential for the SSC membrane with Ti_3_C_2_T_x_ filler in the applications of PEM fuel cells operating at above 100 °C. 

The elastic modulus of the membranes is shown in Figure 9c, in which only the modulus at 80 °C and 100 °C can be displayed because the SSC membrane does not have a clearly elastic area in the stress–strain curve at 120 °C and 140 °C. From Figure 9c, it can be observed that the tensile modulus of the SSC/Ti_3_C_2_T_x_-1.5 composite membrane is higher than the SSC membrane at both 80 °C and 100 °C. The tensile modulus drops significantly when the temperature rises from 80 °C to 100 °C for the SSC membrane, while the modulus of the SSC/Ti_3_C_2_T_x_-1.5 composite membrane decreases much less. The higher modulus of the SSC/Ti_3_C_2_T_x_ composite membrane indicates that the Ti_3_C_2_T_x_ filler can stiffen the SSC polymer matrix and improve the mechanical property. The membranes with high modulus are a desirable material because the membrane will be less deformed or not easily damaged during MEA assembly, especially when a hot press step is necessary. Therefore, it can be concluded that the incorporation of the Ti_3_C_2_T_x_ fillers into the SSC PFSA polymer can strengthen and stiffen the SSC membrane and improve the thermo-mechanical stability of SSC PFSA membranes for potential >100 °C PEM fuel cell applications.

## 4. Conclusions

The SSC/Ti_3_C_2_T_x_ composite membranes with different contents of fewer-layer Ti_3_C_2_T_x_ fillers were successfully prepared by the solution casting method. It was found that the proton conductivity of the composite membranes increased with the Ti_3_C_2_T_x_ filler content up to 1.5 wt% and then decreased with >2.0 wt% filler content, while the hydrogen permeation of the composite membranes displayed the opposite trend with the filler content. Both proton conductivity and hydrogen permeation showed the same turning point at 1.5 wt% Ti_3_C_2_T_x_ filler content. Results from SEM and EDS mapping of Ti element implied that Ti_3_C_2_T_x_ fillers start to aggregate when the filler content is higher than 1.5 wt%, which could be associated with the turning point. 

The proton conductivity of the SSC PFSA membrane could be marginally improved by 15% with the addition of 1.5 wt% Ti_3_C_2_T_x_ filler. While the hydrogen permeation of the composite membrane with 1.5 wt% Ti_3_C_2_T_x_ filler was reduced by 45% compared with that of the pristine SSC membrane. The lower hydrogen permeation could be attributed to the incorporation of the impermeable Ti_3_C_2_T_x_ filler and the decreased hydrophilic ion domain distance examined by the SAXS. 

The thermo-mechanical properties of the SSC/Ti_3_C_2_T_x_ composite membranes were investigated by DMA and tensile testing at the temperature range of 80 °C–140 °C. The Tα of the prepared SSC membranes increased from 123 °C to 149 °C with the increase of the filler content from 0 wt% to 3.0 wt%, which indicated the glass transition temperature of the SSC PFSA membrane can be raised by the Ti_3_C_2_T_x_ filler. The raised glass transition temperature could result in improved mechanical strength at a temperature higher than 100 °C. Therefore, the measured tensile strength of the SSC/Ti_3_C_2_T_x_-1.5 composite membrane was stronger than that of the pristine SSC membrane at 120 °C and 140 °C. 

The higher proton conductivity, the significantly reduced hydrogen permeation, and the improved thermo-mechanical property demonstrated that incorporating 2D Ti_3_C_2_T_x_ (MXene) inorganic filler into the SSC polymer could efficiently improve some membrane properties relevant to the PEM fuel cells operating at 100 °C or higher temperatures for the application of medium- and heavy-duty transportation.

## Data Availability

Not applicable.

## Supplementary Materials

The following are available online at https://www.mdpi.com/article/10.3390/ma14247875/s1, Figure S1: Deconvoluted high-resolution XPS spectra of (a) O 1s and (b) F 1s of Ti_3_C_2_T_x_.
