# Supplementary material for: Improved Thermo-Mechanical Properties and Reduced Hydrogen Permeation of Short Side-Chain Perfluorosulfonic Acid Membranes Doped with Ti3C2Tx
Source: Materials (Basel). 2021 Dec 19;14(24):7875. doi: 10.3390/ma14247875 (PMC8703456; doi:10.3390/ma14247875)
Supplement: Supplementary file 1 [file materials-14-07875-s001.zip › materials-1492216-supplementary (1)--revised.pdf]

materials

MDPI

Article

Improved Thermo-Mechanical Properties and Reduced Hydrogen Permeation of Short Side-Chain Perfluorosulfonic Acid Membranes Doped with $Ti_3C_2T_x$

Panpan Guan [1], Jianlong Lei [1], Yecheng Zou [2] and Yongming Zhang [1,3,*]

1 Center of Hydrogen Science, School of Chemistry and Chemical Engineering, Shanghai Jiao Tong University, Shanghai 200240, China; guanpanpan@sjtu.edu.cn (P.G.); ljl2011@126.com (J.L.)
2 Dongyue Future Hydrogen Energy Materials Company, Zibo 256401, China; yechengzou@dongyuechem.com
3 State Key Laboratory of Fluorinated Functional Membrane Materials, Zibo 256401, China
* Correspondence: ymzhang@sjtu.edu.cn

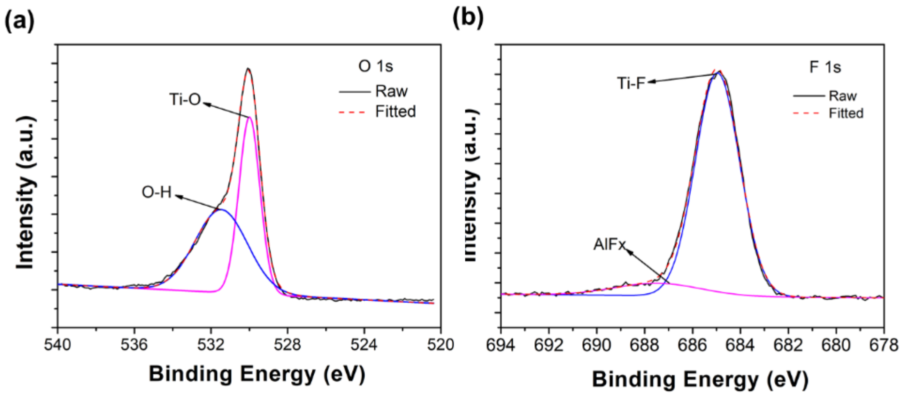

Figure S1. Deconvoluted high-resolution XPS spectra of (**a**) O 1s and (**b**) F 1s of $Ti_3C_2T_x$.
